# Evaluation of phytochemical constituents and sedative-hypnotic activity of the methanol leaf extract of *Ficus exasperata* in mice

**DOI:** 10.14202/vetworld.2019.830-833

**Published:** 2019-06-17

**Authors:** Hudu Garba Mikail, David Dezi Akumka, Mohammed Adamu, Aishatu Ummi Zaifada

**Affiliations:** 1 Department of Pharmacology and Toxicology, Faculty of Veterinary Medicine, University of Abuja, Abuja, Nigeria; 2 Veterinary Teaching Hospital, University of Abuja, Abuja, Nigeria

**Keywords:** *Ficus exasperata*, methanol leaf extract, mice, sedative-hypnotic activity

## Abstract

**Background and Aim:**

Sedative drugs mostly cause dose-dependent depression of the central nervous system which results in hypnosis and anesthesia possibly; however, these agents are associated with some side effects ranging respiratory, digestive, immune system dysfunctions, tolerance, cognitive function deterioration, and physical dependence; hence, investigations of newer and safer agents are, therefore, imperative. The current study was aimed at investigating the sedative-hypnotic (S-H) effects of the methanol leaf extract of *Ficus exasperata* in mice.

**Materials and Methods:**

Phytochemical screening of the leaf extract was conducted, and S-H activity of the plant extract was evaluated. Twenty Swiss Albino mice were randomly divided into four groups of five mice each. The mice in Groups A and B were injected with the extract intraperitoneally (IP) at the dose rate of 100 and 200 mg/kg, respectively, those in Group C were injected with xylazine at the dose rate of 10 mg/kg, while Group D mice received distilled water at the dose rate of 2 ml/kg. All the four experimental groups were injected with ketamine (IP) at the dose rate of 100 mg/kg after 30 min.

**Results:**

Phytochemical analysis of the extract revealed the presence of carbohydrates, cardiac glycosides, reducing sugars, steroids and triterpenes, saponins, tannins, condensed tannins, and flavonoids, while anthraquinones, anthracene derivatives, and alkaloids were absent. Results from the S-H evaluation show no significant difference (p≥0.05) on the onset of sleep time between the four experimental groups; however, statistically significant difference (p≤0.05) was recorded in the sleep duration time between the groups treated with only ketamine and the other experimental groups pre-treated with either the extract or xylazine before ketamine administration. The group pre-treated with a high dose of the plant extract (200 mg/kg) and the treated with ketamine after 30 min exhibited longer sleeping duration time. The plant extract, xylazine and ketamine, sedated the mice for some period of time after arousal from sleep.

**Conclusion:**

Our finding suggests that methanol leaf extract of *F. exasperata* possesses S-H potential that may require further scientific investigations.

## Introduction

Anxiolytics or sedative drugs should reduce anxiety with little or no effect on mental or motor function. Most of these agents’ cause dose-dependent depression of the central nervous system (CNS), leading to sleep-inducing effects (hypnosis) and possibly anesthesia [1]. However, these agents are associated with some side effects ranging from respiratory, digestive, immune system dysfunctions, tolerance, cognitive function deterioration, and physical dependence [2]. *Ficus exasperata* is a well-known tree with very rough leaves used widely in the Nigerian ecoregions; industrially, sandpaper leaves are used in woods polishing [3]. The *Ficus* genus is distinguished by its highly characteristic fruits and often recognized by the milky juice, the minute unisexual flowers often arranged on various shaped receptacles and the prominent stipule that leaves a scar on falling [4]. Different parts of *F. exasperata* Vahl. (Moraceae) are used in traditional medicine as diuretic, analgesic, antiarthritic, antiparasitic, wound healing, vermifuge, ecbolics, abortifacient, and for treating venereal diseases and hemorrhoids. The plant parts are also used as animal fodder [5].

Dissociative anesthetics such as ketamine are likely the most widely used class of anesthetics in veterinary medicine [1]. These agents are associated with emergence delirium (e.g., anxiousness, vocalization, and thrashing). Emergence delirium could be dangerous in larger animals like horses but merely unpleasant in smaller animals. Combine administration of dissociative anesthetics with sedatives or tranquilizers agents such as xylazine, diazepam, and acepromazine can minimize or prevent this unwanted side effect [6]. Thus, the investigation of newer and safer agents with little or no side effects is, therefore, imperative.

Ethnobotanically, the plant *F. exasperata* has been reported to have diverse medicinal uses in treating hemorrhoid, cough, high blood pressure, etc. [3]. Some communities locally use it as a calming agent (personal communications, 2017). The current study was aimed at investigating the sedative-hypnotic (S-H) effects of the methanol leaf extract of *F. exasperata* in mice.

## Materials and Methods

### Ethical approval

This experiment was approved by the University of Abuja Ethical Committee on Animal Use (UAECAU/2018/008), Abuja, Nigeria.

### Study area

The investigation was conducted at the Faculty of Veterinary Medicine, University of Abuja, Nigeria.

### Plant material

The freshly collected plant was identified and a voucher specimen numbered 0516 was deposited at the departmental herbarium. The identification was done by Mr. US Gallah of National Research Institute of Chemical Technology, Zaria, Kaduna State, Nigeria.

### Processing and extraction

Leaf of *F. exasperata* was under the shade air dried for 2 weeks and mechanically pounded into fine particles using pestle and mortar. About 500 g of the pounded plant materials were weighed and extracted by maceration for 72 h in absolute methanol [7]. The extracts were then filtered, evaporated to dryness, and stored in capped bottles inside the refrigerator at 4°C until required.

### Phytochemical analysis

The screening of the phytochemical constituents of the leaf extract was conducted according to standard procedures; the method of Evans [8] was used for the determination of carbohydrates, reducing sugars, anthraquinones, anthracene derivatives, cardiac glycosides, saponins, tannins, condensed tannins, and alkaloids. Flavonoids as described by Silva *et al*. [9], triterpene and steroids as described by Harborne [10] and Sofowora [11]. The phytochemical analysis was conducted at the Faculty of Pharmaceutical Sciences, Pharmacognosy Laboratory of Ahmadu Bello University, Zaria, Nigeria.

### Experimental animals

Swiss Albino mice weighing between 17 and 29 g were used for the experiment; the animals were divided randomly into four groups (A-D) of five mice each totally 20 in number. The mice were marked using picric acid solution on the tail, back, head, and right leg, the fifth mouse was unmarked for easy identification. The animals were kept in cages measuring 44 cm × 28 × 12.5 cm and were allowed access to pelleted feed and tap water *ad libitum*.

### Drugs

Ketamine hydrochloride injection USP (50 mg/ ml), Rotexmedica, Trittau, Germany and Xylazine hydrochloride (20 mg/ml), XYL-M2 injection solution, VMD, Belgium, were used during the experiment.

### Drugs reconstitution

Xylazine 0.1 ml was diluted with 1.9 ml of distilled water to get a solution of 1 mg/ml. Ketamine 0.4 ml was diluted with 1.6 ml of distilled water to get a solution of 10 mg/ml. Forty and 20 mg of the methanol leaf extract were dissolved in 4 ml of distilled water each to get solutions of 10 mg/ml and 5 mg/ml, respectively.

### Treatment of experimental groups

Twenty Swiss Albino mice were used, the mice in Groups A and B were injected intraperitoneally (IP) with the leaf extract at the dose rate of 100 and 200 mg/kg, respectively, Group C mice were injected (IP) with xylazine at the dose rate of 10 mg/kg, while Group D mice received distilled water at dose rate of 2 ml/kg. All the animals in the four experimental Groups A-D were injected with ketamine at the dose rate of 100 mg/kg (IP) after 30 min.

### Assessment of S-H effects

Modified methods of S-H activities screening were employed. Activities such as spontaneous movement [12] or movement in response to catch, rearing (when mouse’s body inclined vertically with hind paws on the floor and forepaws on the wall of the cage [13] or when the mouse stands on its hind paws stretching the forepaws up), and climbing the wire roofing of the cage were used to as an index S-H effects of the drugs and/or extract administered. The activities were measured with some modifications by considering only the presence of these activities was recorded rather than their durations. Mice in each group were observed at 0, 30, 40, 50, 60, 90, and 120 min for 3 min for the presence of these activities, onset and duration of sleep for each mouse was recorded. Response to the drug/extract administration was graded as follows:

1. Sleeping: Laterally recumbent and inactive (no movement at all)
2. Awake: Sitting on four paws and inactive (no movement at all)
3. Slightly Active: Awake and moving slowly in response to catch
4. Active: Awake, moving in response to catch and rearing
5. Very active: Awake, moving in response to catch, rearing, and climbing the wired cage roofing.
  Animals on Grades 2 and 3 were considered sedated and slightly sedated, respectively, while those on Grades 4 and 5 were considered fully awake.

### Statistical analysis

One-way analysis of variance was used to get a level of significance in the data obtained from the study which was followed by Tukey *post hoc* using SPSS statistical software, version 4.0 (IBM, USA). Values ≤0.05 were considered statistically significant. Results are presented as mean plus/minus standard error of the mean.

## Results

**Figure-1:**
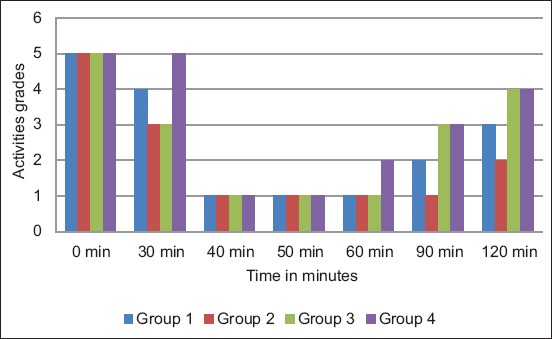
Sedative-hypnotic activities of the methanol leaf extract of *Ficus exasperata* in mice.

The result from the phytochemical analysis revealed the presence of carbohydrates, cardiac glycosides, reducing sugars, steroids and triterpenes, saponins, tannins condensed tannins, and flavonoids, whereas the absence of anthraquinones, anthracene derivatives, and alkaloids was recorded (Table-1). No significant difference (p≥0.05) was recorded in the onset of sleep time between the four different experimental groups; however, there was statistically significant difference (p≤0.05) in the sleep duration time between the group treated with only ketamine and the other three groups that were pre-treated treated with low and high doses of the plant extract and xylazine, respectively, before ketamine administration after 30 min (Table-2). The group pre-treated with high dose of the plant extract (200 mg/kg), then treated with ketamine after 30 min exhibited longer sleeping duration time, followed by the group pre-treated with low dose of the plant extract, then the xylazine pre-treated group, whereas the group treated with only ketamine exhibited the least sleep duration time (Table-2). The plant extract, xylazine and ketamine, calmed the mice by reducing their activities and putting them in sedative position for some period of time after arousal from sleep (Figure-1).

**Table-1:** Phytochemical constituents of the methanol leaf extract of *Ficus exasperate.*

Constituents	Inference
Carbohydrates	+
Cardiac glycosides	+
Reducing sugars	+
Steroid and triterpenes	+
Anthraquinones	−
Anthracene derivatives	−
Saponins	+
Tannins	+
Condensed tannins	+
Flavonoids	+
Alkaloids	−

**Table-2:** Effect of the methanol leaf extract of *Ficus exasperata* on ketamine-induced sleeping time in mice.

Group	Treatment	Onset of sleep (min)	Duration of sleep (min)
Group 1	100 mg/kg extract+ketamine 100 mg/kg after 30 min	3.400±0.200	45.8000±0.37*
Group 2	200 mg/kg extract+ketamine 100 mg/kg after 30 min	2.800±0.200	71.2000±9.25^a^*
Group 3	10 mg/kg xylazine+ketamine 100 mg/kg after 30 min	3.000±0.320	35.4000±3.85^a^*
Group 4	100 m/kg ketamine only after 30 min	3.000±0.320	20.8000±2.98*

* Significant difference (p≤0.05) between Group 4 and Groups 1, 2, and 3,

a significant difference (p≤0.05) between Groups 2 and 3

### Activities grade key

(1) Sleeping, (2) awake but sedated, (3) awake but slightly sedated active, (4) awake and active, and (5) awake and very active.

## Discussion

In the present study, chemical constituents recorded present in the methanol leaf extract of *F. exasperata* agrees with those reported by Adebayo *et al*. [14] and are slightly at variance with those reported by Lawal *et al*. [3] present on stem bark extract of the plant. Flavonoids and saponins have been shown to have S-H effects [12,15]. The S-H effects of *Ficus abutilifolia* methanol leaf extract found in this study could, therefore, be linked to the presence of phytochemical constituents such as flavonoids and saponins.

The activities of the mice were affected by either the drug or extract administration, the animals were very active before receiving any form of treatment; however, their activities subsided, i.e., they were sedated following treatment with either the extract or xylazine and hypnotized finally due to ketamine administration. Movement of any form was not seen in the hypnotized mice except that of the abdominal muscle indicating evidence of respiration. The mice did not resume their very active position up to the end of the experiment. These findings are in line with the reports of Moniruzzaman *et al*. [16] and Khan *et al*. [17] regarding the S-H activity of natural products of plant origin. Mostly, the S-H effect of drugs is dose-dependent, with sedation initially then followed by hypnosis and possibly anesthesia with increasing doses of the agent in use and visa vis [1].

Xylazine and leaf extract pre-treatment prolong the sleeping time in comparison with ketamine treatment alone, the prolongation dose-dependent as the high extract dose produced the longest sleeping time than the low dose. Similarly, all the administered agents induced a calming effect on the mice, especially after arousal from sleep, this effect is otherwise known as sedation which lasts for some period of time before the resumption of normal activities by the treated mice. Thus, the leaf extract of *F. exasperata* possesses S-H activity.

The mechanism by which the plant stem bark extract produced the S-H effect was not investigated; however, most S-H drugs facilitate the actions of GABA, a major inhibitory transmitter in the CNS [1]. GABA_A_ receptor activation leads to increased C1 ion influx; GABA_B_ receptor activation causes increased efflux of K^+^. Both mechanisms result in membrane hyperpolarization [1]. Dissociative anesthetic drugs prevent the binding of glutamate, the major excitatory neurotransmitter in the CNS to the N-methyl-D-aspartate receptor, resulting in depressed activities of thalamocortical and limbic system as well as the activities of the reticular activating systems nuclei [6].

## Conclusion

Our current finding suggests that methanol leaf extract of *F. exasperata* possesses S-H activity that may require further investigations scientifically to find out the active ingredient and mechanism of action contained in the plant extract.

## Authors’ Contributions

HGM: Designed and wrote the manuscript; HGM, DDA, MA, and AUZ: Conducted the research. All authors read and approved the final manuscript.
